# Attempts to Develop Vaccines Against Alzheimer’s Disease: A Systematic Review of Ongoing and Completed Vaccination Trials in Humans

**DOI:** 10.7759/cureus.40138

**Published:** 2023-06-08

**Authors:** Ajit Thakur, Sunil Bogati, Sagar Pandey

**Affiliations:** 1 Internal Medicine, B.P. Koirala Institute of Health Sciences, Dharan, NPL; 2 Internal Medicine, One Brooklyn Health System Interfaith Medical Center, Brooklyn, USA

**Keywords:** immunotherapy, randomized controlled trial (rct), behavioral neurology, alzheimer's disease, vaccine

## Abstract

In this systematic review, we evaluate the safety, tolerability, and immunogenicity of vaccination efforts against Alzheimer’s disease (AD) in human subjects from both ongoing and completed vaccination trials. Databases like PubMed, Embase, and Scopus were used to identify relevant articles on completed vaccination trials whereas the clinicaltrials.gov database was used for identifying ongoing clinical trials for vaccination against AD in humans until January 2022. Only interventional randomized or non-randomized clinical trials which reported on the safety and immunogenicity of vaccine against AD in humans were included. Cochrane risk of bias tool-2 (RoB-2) or risk of bias in non-randomized studies- of intervention (ROBINS-I) was used for risk of bias assessment as appropriate. A narrative descriptive synthesis of the findings was done. Sixteen randomized/non-randomized clinical trials (phase I: six and phase II: 10) for seven different types of vaccines against AD were identified comprising a total of 2080 participants. Apart from the development of meningoencephalitis in 6% of patients receiving AN1792 in an interrupted phase II trial, the rest of the trial reported promising results on the safety and immunogenicity of vaccines. While only a subset of reported adverse events was treatment related, none of the fatalities reported during the trial were considered related to vaccine administration. The serological response rate ranged from 100% (4/16 trials) to 19.7% in an interrupted trial. Although current trials show promising results, adequately powered phase III studies are needed to conclusively establish the safety, immunogenicity and therapeutic efficacy of vaccines.

## Introduction and background

Alzheimer’s disease (AD), the leading cause of dementia in the elderly, is a neurodegenerative disorder believed to be due to the deposition of amyloid plaques and abnormal tau protein in the brain [1]. Over 50 million people are estimated to be living with dementia worldwide in 2020 with more than half (60%) of the patient population belonging to low- and middle-income countries. In addition, the number of patients living with dementia is projected to double every 20 years, reaching a staggering number of 152 million by 2050 [2]. Available treatment options for AD include cholinesterase inhibitors (like donepezil, rivastigmine, and galantamine), N-methyl-D-aspartate (NMDA) receptor antagonists (like memantine), and monoclonal antibodies against amyloid beta-like aducanumab [3,4]. Although currently, available treatment options for AD have a modest therapeutic effect on neuropsychiatric and functional outcomes, any conclusive evidence on stopping the progression of AD or even decreasing the amyloid burden to achieve symptomatic recovery is lacking [5-8].

On the other hand, efforts to shift the paradigm of management of AD from tertiary prevention to primary preventive approaches could help reduce the burden of AD in the future. Novel active and passive immunotherapeutic approaches are, therefore, being actively explored as potential preventive measures against AD [9-11]. Active immunotherapeutic approaches targeting Aß or tau-related pathologies in transgenic mice have shown promising outcomes in terms of reduction in the extent and progression of AD-like pathologies, reducing inflammation, improving cognitive performance, and reducing memory loss [12-16]. This has led to the progression of preclinical studies in transgenic mouse models to clinical trials in human subjects to evaluate the safety, tolerability, and immunogenicity of active immunotherapy. However, a systematic literature review and qualitative analysis of safety, tolerability, and immunogenicity of early-phase clinical trials, both ongoing and completed, for active immunotherapeutic efforts against AD is lacking. This study, therefore, is done to systematically review the safety, tolerability, and immunogenicity of vaccination efforts against AD in human subjects from randomized or non-randomized clinical trials.

## Review

Methods

Protocol and Registration

We conducted this review following preferred reporting items for systematic review and meta-analysis (PRISMA) guidelines [17]. PRISMA checklist is available in S1 File. The protocol was drafted and registered before conducting the systematic review in the international prospective register for systematic review (PROSPERO) with registration number: CRD42022299172 which is available in S2 File.

Inclusion Criteria

We included peer-reviewed published full-text articles which assessed the safety and immunogenicity of vaccines against AD in human beings. Only interventional randomized or non-randomized clinical trials published in the English language were included.

Exclusion Criteria

Clinical trials for vaccines against AD among non-human or animal models were not included. In addition, clinical trials evaluating the safety and efficacy of passive immunotherapy like monoclonal antibodies, drug therapies, interventions like deep brain intervention techniques, etc. against AD in humans or animal models were excluded. Prospective or retrospective cohort studies, case-control studies, review articles, editorials, case reports, and case series were also excluded.

Search Strategy

We conducted a systematic search for relevant articles in databases like PubMed, Embase, and Scopus. We also searched the clinicaltrials.gov database for identifying relevant ongoing clinical trials for vaccination against AD in humans. A free search was conducted without any predetermined timeframe for published studies. The search strategy included keywords like “Alzheimer*” and “vaccine” combined with the Boolean operator “AND”. Only full-text articles published in the English language were included. The last search was conducted in January 2022.

Data Screening and Extraction

AT, SB, and SP independently screened and retrieved the articles using the systematic search strategy. Studies were reviewed for eligibility by a screening of titles and abstracts followed by full-text screening. AT, SB, and SP cross-examined the search results to check for their decisions. Upon disagreement, a careful review of the inclusion and exclusion criteria was the basis of the final decision. The principal investigator (AT) made the final decision after thorough reviewing when an agreement could not be reached. Zotero, a research tool to collect, organize, and manage research publications was used to keep a record, and remove duplicates.

Studies retrieved from a systematic search were imported to Zotero. Screened studies were placed into appropriate subfolders created in Zotero based on the decision to include or exclude them. Final data were extracted from the included studies. The following information was extracted from included studies (if available): type of study, vaccine characteristics, route of administration, a dosing schedule of the vaccine, inclusion, and exclusion characteristics for participants in vaccine trials, number of participants both in vaccination and control arms, duration of follow up, reported adverse events, tolerability and immunogenicity of the vaccine, etc. All records were entered into an Excel spreadsheet. AT, SB, and SP independently extracted the data and both authors cross-checked the extracted data in an alternate fashion.

Risk of Bias

AT, SB, and SP independently assessed the risk of bias in included studies, and disagreements between the authors were resolved by further discussion. 

The risk of bias was assessed using the revised Cochrane risk of bias tool-2 (RoB-2) for RCTs [18]. RoB-2 consists of five key domains which assess bias based on aspects of trial design, conduct, and reporting. Domain-level risk of bias and subsequent overall risk of bias judgment was generated by an algorithm based on answers to the signaling questions on each domain. Risk of bias judgment was categorized as “low risk of bias,” “high risk of bias” or risk of bias expressing some concerns.”

The risk of bias for non-randomized interventional clinical trials was evaluated using the Risk of Bias in Non-randomized Studies- of Intervention (ROBINS-I) assessment tool [19]. It evaluated the risk of bias under seven domains namely bias due to confounding, bias due to selection of participants into the study, bias in classification of interventions, bias due to deviations from intended interventions, bias due to missing data, bias in measurement of outcomes, and bias in selection of reported results. The judgment on risk of bias was made based on the answers to the signaling questions. Both domain level and overall risk of bias judgment were categorized as low/ moderate/serious/critical risk of bias and no information.

Data Analysis and Data Synthesis

A narrative descriptive synthesis of the findings was done. The results of this review were primarily focused on summarizing and reporting the safety, tolerability, and immunogenicity of vaccines against AD in various clinical trials. Safety of the vaccines was reported in terms of the reported number of adverse events along with adverse events considered related to study treatment and a number of reported fatalities. Tolerability to the vaccines was reported in terms of a number of participants who dropped out of the trial due to adverse events. Lastly, immunogenicity was reported in terms of serological response rate and/or antibody titers against the antigen component of the vaccine.

Results

Study Selection

**Figure 1:**
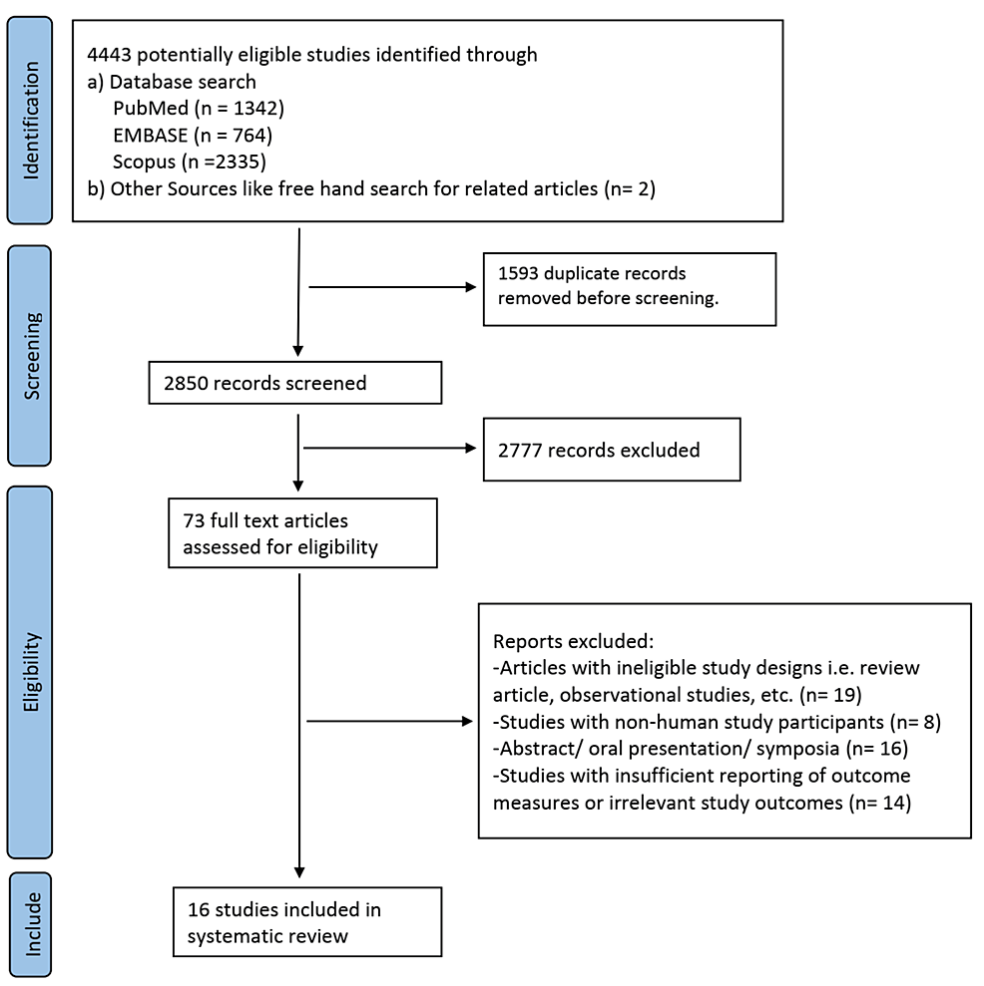
PRISMA flow diagram for study selection PRISMA: Preferred Reporting Items for Systematic Reviews and Meta-Analyses.

We identified a total of 4,443 potentially eligible studies through a combination of database search and free hand search for related articles. After excluding 1,593 duplicate records, 2,850 articles were screened for inclusion via title and abstract screening. This yielded 73 articles for full-text screening after excluding 2,777 articles that failed to address the review question. Finally, 16 studies were included in the systematic review after full-text screening of the articles. The remaining 57 articles were excluded from the review due to the reasons mentioned in the PRISMA flow diagram in Figure 1.

Characteristics of Included Studies

Out of the 16 included studies [20-35], six were phase I clinical trials, ten were phase II clinical trials, and comprised a total of 2080 participants. The earliest phase I trial with human aggregated Aß42 (AN1792) by Bayer et al. was conducted in 2000 in the UK across four centers to evaluate the safety, tolerability, and immunogenicity of the vaccine with 80 trial participants [20]. Several clinical trials with different AD vaccine designs were conducted in subsequent years. Among the 16 included studies in this review, four are randomized, double-blind placebo-controlled phase I clinical trials, two are open-label single arm phase I interventional clinical trials, nine are randomized double blind/third party unblinded placebo-controlled phase II clinical trials and one is open label single arm phase II clinical trial. A total of five out of 16 included studies evaluated the safety, tolerability and other exploratory endpoints among patients receiving Vanutide Cridificar (ACC-001) in phase II clinical trials. Potential safety and tolerability of CAD106 as vaccine against AD was explored in one phase I and two phase II clinical trials. Likewise, vaccines like AN1792, AADvac1, UB-311, ABvac40 and AD02 were the candidates for vaccine against AD in rest of the studies. A summary of the characteristics of the included studies is presented in Table 1.

**Table 1:** Characteristics of included studies a: Intramuscular; b: Subcutaneous

Study ID, Name of vaccine	Study Design	Recruitment dates	Route of administration, Dosing Schedule of vaccine	Study participants	Number of study participants (Vaccine arm/ Control arm)	Duration of the study
Phase I Studies						
Bayer 2005, AN1792 [20]	Randomized, double blind, placebo-controlled phase I study	April, 2000 to June, 2002	IM^a^, Four doses on day 0 and weeks 4, 12, and 24. Additional injections at weeks 36, 48, 60 and 72 for patients entering extension phase.	Patients aged ≤85 years with mild to moderate AD	80 (64/16)	84 weeks
Winblad 2012, CAD106 [21]	Randomized, double blind, placebo-controlled phase I study	August, 2005 to March, 2008	SC^b^, Three doses at week 0, 6 and 18 in cohort 1 and week 0, 2 and 6 in cohort 2	Patients aged 50-80 years with mild to moderate AD	58 (46/12)	52 week study period with 2 year safety follow-up phase
Novak 2016, AADvac1 [22]	Randomized, double blind, placebo-controlled phase I study	June, 2013 to March, 2015	SC^b^, Vaccine arm: Six doses, once every four weeks Control arm: Three doses in open label extension phase	Patients aged 50-85 years with mild to moderate AD	30 (24/6)	12 week study period with 12 weeks of open label extension period
Wang 2017, UB-311 [23]	Open label phase I single arm interventional clinical trial	Feb, 2009 to April, 2011	IM^a^, Three doses at 0, 4 and 12 weeks.	Patients aged 50-85 years with mild to moderate AD	19 (19/NA)	24 week interventional study with 24 week observational extension study
Lacosta 2018, ABvac40 [24]	Randomized, double blind, placebo-controlled phase I study	December, 2013 to March, 2015	SC^b^, Two (as per initial protocol) or three (as per amended protocol) doses of vaccine at 4 weeks interval.	Patients aged 50-85 years with mild to moderate AD	24 (16/8)	12-16 weeks of interventional study with one year of safety follow-up
Novak 2018, AADvac1 [25]	Open label phase I single arm interventional follow-up clinical trial	March, 2014 to August, 2016	SC^b^, Patients in the vaccine arm of the parent trial: two booster doses at 24 weeks interval (with first booster given only if the antibody titer fall below 75% and obligatory second booster) -Patients in the placebo arm of parent trial: three doses of the vaccine at four weeks interval and subsequently entered above-described booster regimen.	Patients who had completed the preceding phase I study of AADvac1	26 (26/NA)	72 weeks follow-up
Phase II Studies						
Gilman 2005, AN1792 [26]	Randomized, double blind, placebo-controlled phase IIa study	September, 2001 and December, 2002	IM^a^, Day 0 and at months 1, 3, 6, 9, and 12. Due to premature discontinuation, patients received only one to three injections.	Patients aged 50-85 years with mild to moderate AD	372 (299/73)	15 months
Arai 2015, Vanutide Cridificar (ACC-001) [27]	Randomized, third-party unblinded placebo-controlled multiple ascending dose phase IIa study	Study 1: December, 2008 to July, 2012 Study 2: August, 2009 to January, 2013	IM^a^, Study 1: Day 1 and weeks 12, 26, 36 and 52. Study 2: Day 1 and weeks 4, 12, 26, and 52	Patients aged 50-85 years with mild to moderate AD	Study 1: 40(30/10) Study 2: 32(24/8)	52 weeks dosing period with an additional 52 weeks follow up period
Farlow, 2015, CAD106 [28]	Two randomized, double blind, placebo-controlled phase IIa core studies (2201;2202) and their respective two open label extension studies (2201E;2202E)	Core study 2201: July 2008 to Feb 2010 Core study 2202: October 2008 to Nov 2010 Extension study 2201E: Sept 2009 to July 2011 Extension study 2202E: Dec 2009 to Feb 2012	SC^b^/IM^a^, For core study 2201: Three injections at weeks 0, 6 and 12 For core study 2202: Three injections at weeks 0, 2 and 6. For extension study: Four injections at 12 weeks interval	For core studies: Patients aged 40 to 85 years with mild AD For extension studies: Patients who completed the core studies without major safety concerns	Core study 2201: 27(22/5) Core study 2202: 31(25/6) Extension study 2201E: 21(21/NA) Extension study 2202E: 24(24/NA)	52 weeks for core studies and 66 weeks for their respective open label extension studies
Schneeb-erger 2015, AD02 [29]	Randomized, double blind, placebo-controlled phase II study	01 October, 2010 to 06 December, 2013	SC^b^, Six injections at weeks 0, 4, 8, 12, 40, and 65.	Patients aged 50-80 years with early AD	332 (233/99)	65 weeks
Ketter 2016, Vanutide Cridificar (ACC-001) [30]	Randomized, double blind, placebo-controlled phase II study	February, 2011 to January, 2014	IM^a^, A total of six injections on day 1 and at weeks 4, 12, 26, 52, and 78	Patients aged 50-89 years with mild to moderate AD	126 (86/40)	104 weeks (78 weeks of interventional study with an additional six months follow up)
Pasquier 2016, Vanutide Cridificar (ACC-001) [31]	Randomized, third-party unblinded placebo-controlled multiple ascending dose phase IIa study	May, 2007 to February, 2013	IM^a^, Day 1, and at months 1, 3, 6, and 12.	Patients aged 50-85 years with mild to moderate AD	245 (184/61)	12 months dosing period with an additional 12 months of follow up
van Dyck 2016, Vanutide Cridificar (ACC-001) [32]	Randomized, third-party unblinded, placebo-controlled phase II study	February, 2011 to February, 2014	IM^a^, A total of six injections on day 1 and at weeks 4, 12, 26, 52, and 78.	Patients aged 50-85 years with early AD	63 (42/21)	104 weeks (78 weeks of interventional study with an additional six months follow up)
Hüll 2017, Vanutide Cridificar (ACC -001) [33]	Third party unblinded (EU/US studies) or open label (Japan study) single arm phase IIa long-term extension study	EU/US Studies: June 15, 2009 to December 19, 2013 Japan Study: December 20, 2010 to December 13, 2013	IM^a^, Day 1 and month 6, 12 and 18.	Patients who participated in 1 of 4 placebo controlled, multiple-ascending-dose parent studies (EU, US, and 2 in Japan) and completed parent study week 78 (week 104 for Japan study cohort 1), received at least 3 doses of investigational product, and had a Mini-Mental State Examination (MMSE) score ≥10 at extension study screening	EU/US Studies: 160 (160/NA) Japan Study: 53(53/NA)	18 months dosing period with an additional 6 months of follow up
Vandenb-erghe 2017, CAD106 [34]	Randomized, double blind, placebo-controlled phase IIb study	March, 2010 to December, 2012	IM^a^, Upto seven injections at weeks 0, 6, 12, 24, 36, 48, and 60.	Patients aged ˂85 years with mild AD	121 (106/15)	60 weeks of dosing period with follow up until 90 weeks
Novak 2021, AADvac1 [35]	Randomized, double blind, placebo-controlled phase II study	16 June, 2016 to 30 May, 2017	SC^b^, Patients received a total of 11 doses, divided into an initial treatment regimen of 6 doses at 4-week intervals, followed by 5 booster doses administered at 14-week intervals.	Patients aged 50-85 years with mild AD	196 (117/79)	104 weeks

Risk of Bias in Included Studies

**Figure 2:**
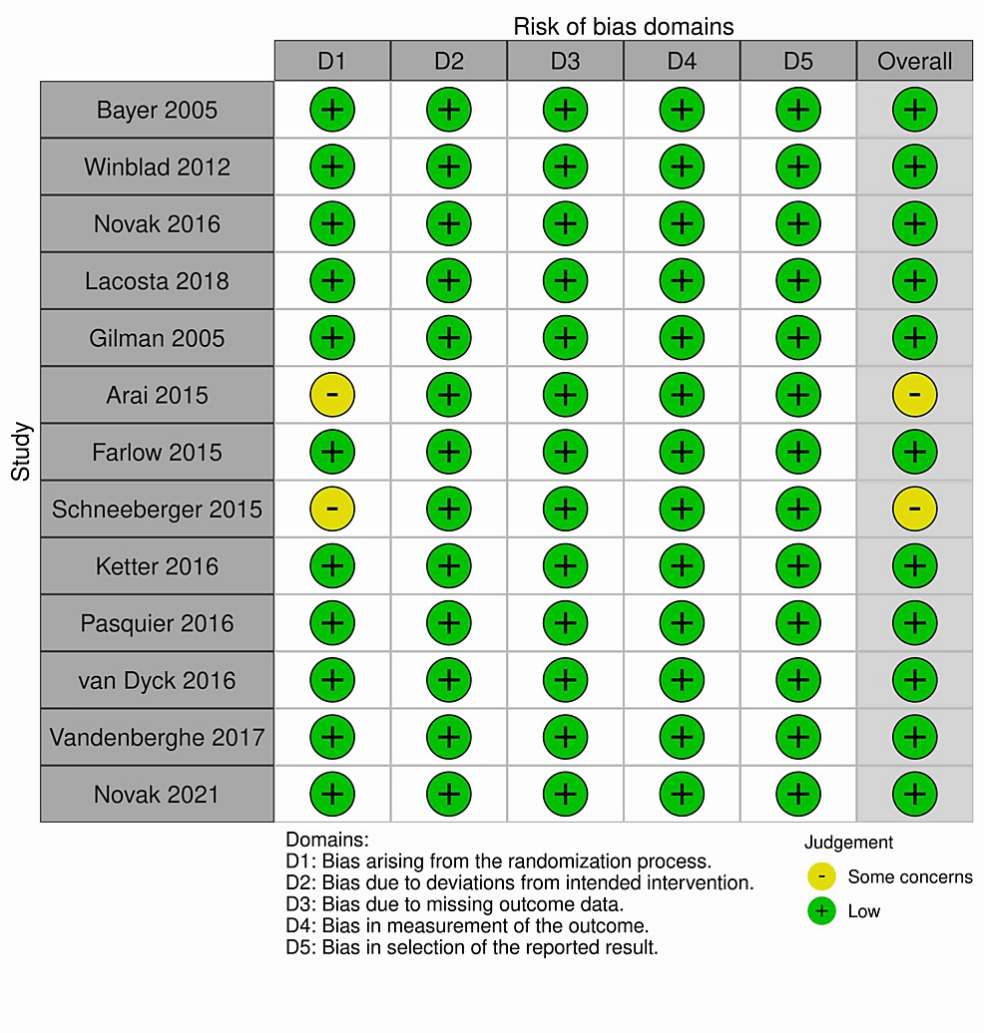
ROB 2 domain level risk of bias judgments for each individual studies Bayer 2005 [20], Winblad 2012 [21], Novak 2016 [22], Lacosta 2018 [24], Gilman 2005 [26], Arai 2015 [27], Farlow 2015 [28], Schneeberger 2015 [29], Ketter 2016 [30], Pasquier 2016 [31], van Dyck 2016 [32], Vandenberghe 2017 [34], Novak 2021 [35]

**Figure 3:**
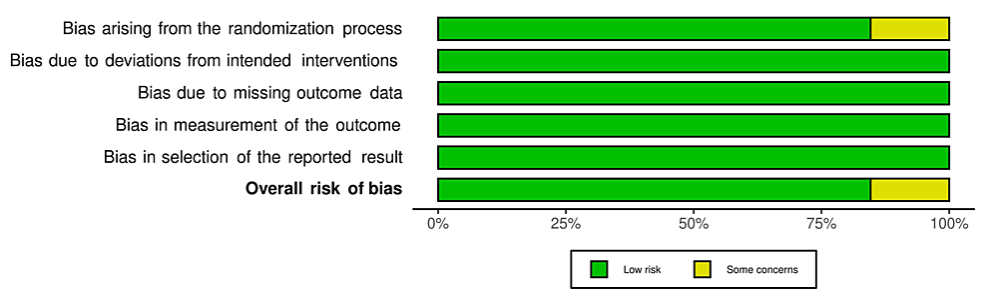
ROB 2 weighted bar plots of risk of bias judgements across each domain

For randomized controlled trials (13 out of 16 studies), revised Cochrane risk of bias tool (ROB 2) evaluated risk of bias for primary outcomes in five key domains, i.e., bias arising from randomization process, bias due to deviation from intended interventions, bias due to missing outcome data, bias in measurement of the outcome and bias in selection of the reported result. All of the studies except the studies by Arai et al. [27] and Schneeberger et al. [29] were found to have low overall risk of bias. Arai et al. and Schneeberger et al., on the other hand, reported no information about the method of randomization and whether or not the allocation sequence was concealed until participants were enrolled and assigned to interventions. This led to risk of bias judgment in domain one and overall risk of bias as “some concern”. All of the randomized controlled trials ensured that participants, carers and people delivering the interventions were unaware of the intervention groups during the trial and an appropriate analysis was used to estimate the effect of assignment to intervention leading to low risk of bias judgment in domain two. In addition, outcome data were available for all, or nearly all, randomized participants resulting in low risk of bias in domain three. Likewise, in domain four, the method of measurement of outcome was not inappropriate and the measurement or ascertainment of the outcome did not differ between intervention groups. Furthermore, the outcome assessors were also unaware of the intervention received by the study participants resulting in low risk of bias. Finally, risk of bias in selection of reported result was low in all studies because they analyzed the data in accordance to pre-specified plan and reported results for the outcome domain corresponding to all intended outcome measurements and analyses. Traffic light plots of the domain level judgments for each individual studies and weighted bar plots of the distribution of risk of bias judgments within each bias domain are presented in Figures 2, 3, respectively.

**Figure 4:**
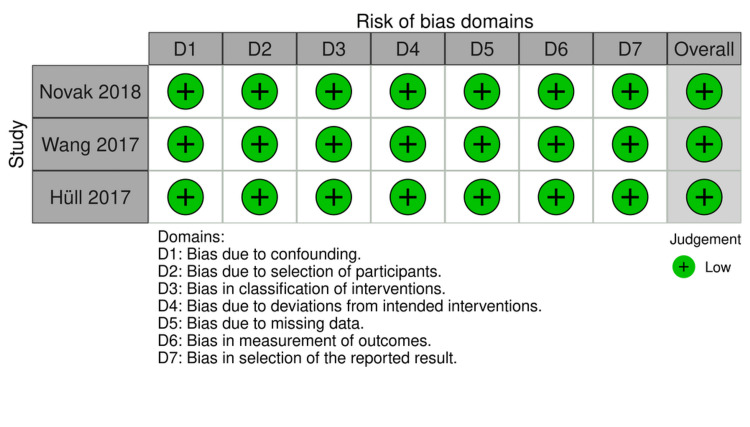
ROBINS-I domain level risk of bias judgements for each individual studies Novak 2018 [25], Wang 2017 [23], Hüll 2017 [33]

**Figure 5:**
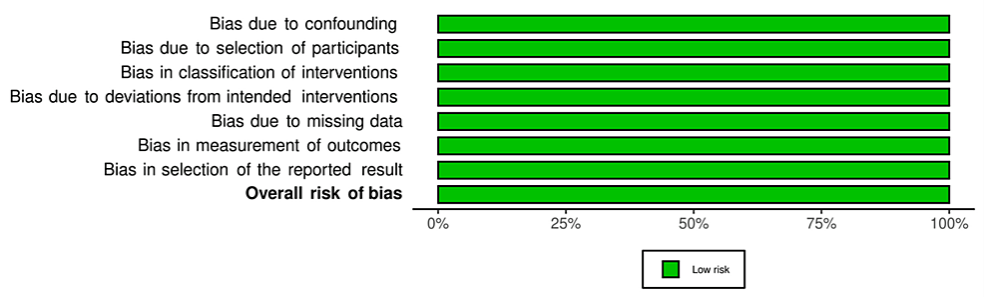
ROBINS-I weighted bar plots of risk of bias judgements across each domain

For non-randomized interventional clinical trials (three out of 16 studies), the risk of bias in non-randomized studies - of intervention (ROBINS-I) assessed risk of bias due to confounding, selection of participants, classification of interventions, deviations from intended interventions, missing data, measurement of outcomes and selection of reported results. The risk of bias was low under all domains resulting in low overall risk of bias. Figures 4, 5 represent traffic light plots of the domain level judgments for each individual studies and weighted bar plots of the distribution of risk of bias judgments within each bias domain, respectively.

Overview of Completed Vaccination Trials

First in human phase I clinical trial for AD vaccine, AN1792 was conducted by Bayer et al. which demonstrated a lack of significant safety concerns for the vaccine [20]. The study, however, reported four cases of nonfatal serious adverse events considered related to the study treatment namely rash, confusion and syncope, encephalitis (T-lymphocyte meningoencephalitis detected on autopsy) and worsening of dementia. In addition, treatment related adverse events like hostility (n = 3) and hallucination (n = 1) lead to discontinuation of trial in four patients, three in vaccine arm and one in placebo arm. The subsequent phase IIa trial for the vaccine was interrupted because of reported cases of meningoencephalitis in 6% of immunized patients, although no indication of its association with the vaccine was reported in prior studies. An induction of T-cell inflammatory response was hypothesized to be the cause behind incidence of meningoencephalitis rather than due to the development of antibodies against human aggregated Aß42 [26].

Safety, tolerability and immunogenicity of novel active Aß immunotherapy, CAD106 was assessed in phase I clinical trial by Winblad et al. [21] which showed a favorable safety profile and an antibody response. Although cases of meningoencephalitis were not reported, two patients had pericerebral meningeal MRI changes attributed due to lumbar puncture or minor head trauma. None of the serious adverse events were considered related to the study treatment. Two phase IIa randomized placebo-controlled trials and their subsequent open label extension studies for CAD 106 conducted by Farlow et al. [28] supported the safety and tolerability profile of the vaccine. A single case of intracerebral hemorrhage (ICH) as a serious adverse event considered related to treatment was seen in core study. In addition, four patients, two of them in cores studies and two in extension studies, presented with amyloid related imaging abnormalities corresponding to micro hemorrhages (ARIA-H). No cases of amyloid related imaging abnormalities corresponding to vasogenic edema (ARIA-E) or meningoencephalitis were reported. Similarly, Vandenberghe et al. [34] in a phase IIb placebo-controlled trial for CAD106 reported a balance between antibody response and tolerability. Three serious adverse events namely allergic dermatitis, atrial fibrillation and acute psychosis seen in vaccine arm were considered related to treatment. MRI findings compatible with ARIA (ARIA-H, n= 5; ARIA-E, n=1) were seen in six patients in vaccine arm; however, no signs or symptoms of CNS inflammation were reported.

Tau vaccine AADvac1 for AD was investigated for its safety, tolerability and immunogenicity in phase I and phase II clinical trials by Novak et al. [22,25,35] In placebo-controlled phase I trial, apart from mild injection site local reactions, viral infection and epileptic seizure were the only two serious adverse events that were considered related to treatment. Furthermore, besides a case of microhemorrhage in one patient with pre-existing microhemorrhage, no cases of meningoencephalitis or amyloid related imaging abnormalities (ARIA-H or ARIA-E) were reported [22]. A 72-week interventional phase I follow-up study of parent trial also found AADvac1 to have a benign safety profile [25]. Clinically silent new micro-hemorrhages were observed in one ApoE4 homozygote, and superficial hemosiderin was found in one ApoE4 heterozygote, consistent with background incidence of such lesions. The subsequent phase II trial also found the vaccine to be safe and well tolerated [35].

Vanutide Cridificar (ACC-001) and its safety, tolerability and immunogenicity in patients with AD was reported in five studies, four of them being placebo controlled randomized phase II trial and one interventional single arm long term extension study [27,30-33]. van Dyck et al. [32] reported that treatment with ACC-001 was not associated with a higher rate of serious adverse events compared with placebo. The study also reported no incidences of immune-mediated adverse events along with any amyloid related imaging abnormalities. Similar conclusions were drawn from two phase II studies by Arai et al. [27]. However, Ketter et al. [30] in a phase II trial reported five cases of ARIA-E in vaccine arm leading to early discontinuation of the study medication; the events were however, considered mild to moderate in severity and their frequency too small to draw any conclusions. Similarly, Pasquier et al. [31] in two phase IIa multiple ascending dose studies reported two cases of ARIA-E, one symptomatic and other asymptomatic, in ACC-001 group. Symptomatic ARIA-E was resolved after treatment with steroids for three days, and asymptomatic ARIA-E resolved without treatment. Hull et al. [33] in single arm interventional extension studies observed similar safety profile as that in parent trials and suggested that side effects did not pose a principal limitation for anti-amyloid immunotherapy. An overview of completed vaccination trials in human is summarized in Table 2.

**Table 2:** Overview of completed vaccination trials for vaccine against AD in humans

Study ID	Vaccine characteristics	Study Outcomes	Adverse events (AEs)	Tolerability	Immunogenicity
Phase I Studies					
Bayer 2005 [20]	Active vaccine against human aggregated Aß42	Safety, tolerability and immunogenicity of vaccine	AEs were reported in 97.5% of patients during the study period. AEs that were considered treatment related were reported in 28.1% of patients in vaccine arm and 6.3% of patients in control group. Five patients (6.3%) four in active treatment and one in control group, died during study period. None of them were considered related to study treatment. Four patients had non-fatal serious AEs considered related to study treatment.	A total of 10 patients, nine in active treatment and one in control group, withdrew from trial due to adverse events.	Of all patients who received AN1792 (n= 64, 53.1% had a positive anti-AN1792 antibody titer at some point during the study. Of those who entered the protocol extension (n= 51), 58.8% of patients had a positive antibody response and 56.9% were considered anti-AN1792 antibody responders at some point during the study. Of the 13 AN1792-treated patients who did not enter the protocol extension, 15.4% were considered anti-AN1792 antibody responders.
Winblad 2012 [21]	An active Aß immunotherapy designed to generate production of antibodies against a small Aß peptide fragment (Aß_1-6_) acting as a B-cell epitope avoiding an Aß-specific T-cell response	Safety, tolerability and Aß-specific antibody response of vaccine	-56/58 patients (97%) reported AEs (45 events in vaccine arm and 11 events in placebo arm). -Nine patients reported serious AEs (eight in vaccine arm and one in placebo arm), none of them considered related to study treatment - No deaths or discontinuation from trial due to AEs.	One patient in cohort 2 withdrew consent at week 26 after receiving all three doses of vaccine	16 of 24 (67%) CAD106-treated patients in cohort one and 18 of 22 (82%) in cohort two developed Aß-IgG antibody response meeting pre-specified responder threshold. One of 12 placebo-treated patients (8%) had Aß-IgG concentrations that reached the responder threshold
Novak 2016 [22]	An active peptide vaccine pathological tau protein	Safety, tolerability and immunogenicity of vaccine	Local injection site reaction in 16/30 vaccinated patients (53%). Five patients who received the vaccine had serious AEs, two of them considered possibly related to treatment. No deaths reported in trial.	2/30 patients discontinued participation in trial because of serious adverse events	29/30 treated patient developed an IgG response at the latest after third dose of vaccine. A geometric mean IgG antibody titer of 1:31415 against pathological tau component was achieved.
Wang 2017 [23]	Synthetic peptide vaccine comprising two Aß_1-14_ targeting peptides (B-cell epitopes) each linked to different helper T-cell peptide epitopes as a chimeric peptide to maximize immunogenicity	Safety, tolerability and immunogenicity of vaccine	Out of 42 adverse events following vaccination, 16 (47.4%) were categorized as treatment related AEs. No deaths reported in trial.	The vaccine was well tolerated in all 19 patients	UB-311 generated over the pre-specified threshold anti- Aß antibody response at week eight after two immunization in all of the 19 AD patients. The immune response peaked at week 16 and remained elevated at week 48.
Lacosta 2018 [24]	An active vaccine against C-terminal end of Aß_40_	Safety, tolerability and immunogenicity of vaccine	Overall, 71 AEs recorded in 18 patients. 7/8 patients (88%) in placebo group and 11/16 patients (69%) in vaccine group suffered at least on adverse event during study. AEs in only two patients in vaccine arm were considered possibly treatment related. No deaths reported in trial.	The vaccine was well tolerated	Eleven of 12 (~92%) individuals receiving three injections of ABvac40 developed specific anti- Aß40 antibodies
Novak 2018 [25]	An active peptide vaccine against pathological tau protein	Long term safety and immunogenicity	Injection site reaction observed in 50% of patients on AADvac1 treatment. Six serious AEs, none judged to be related to vaccination, were seen. No deaths reported in trial.	Four patients discontinued owing to serious adverse events, one for an adverse event and one for non-compliance	All responders retained an IgG antibody response over 6 months without administration, with titers regressing to a median 15.8% of titers attained after the initial six-dose vaccination regimen. Booster doses restored previous IgG levels.
Phase II Studies					
Gilman 2005 [26]	Active vaccine against human aggregated Aß42	Safety, tolerability and immunogenicity of vaccine	AEs were reported in 266/300 (88.7%) patients who received vaccine and 59/72 (81.9%) placebo-treated patients. Treatment-related AEs were reported in 77/300 (25.7%) patients in vaccine arm, of which 24/300 (8%) were reported as severe and the majority of these were associated with encephalitis. Seven patients died during the study follow-up period, five in vaccine and two in control arm.	The trial was prematurely discontinued due to the occurrence of encephalitis in 18/300 (6%) patients in vaccine arm.	Of the 300 patients who received the vaccine, 59 (19.7%) were antibody responders as per the predefined criteria.
Arai 2015 [27]	Active peptide vaccine against N-terminus 1-7 of Aß peptide	Safety, tolerability and immunogenicity of vaccine	Study 1: Treatment emergent AEs were reported in 36/40 participants, majority of them were mild to moderate in severity and AEs in only 14 subjects were considered treatment related. No deaths were reported in the study. Study 2: Treatment emergent AEs were reported in 30/32 participants, majority of them were mild to moderate in severity and AEs in only 17 subjects were considered treatment related. No deaths were reported in the study	Study 1: No withdrawal from the study because of adverse events. Study 2: Three subjects in the vaccine arm withdrew from study because of adverse events.	Treatment groups in both the studies achieved measurable and sustained geometric mean titers of anti-Aß specific IgG antibodies following second immunization. None of the patients in control arm of both the studies achieved measurable anti-Aß specific IgG antibodies.
Farlow 2015 [28]	An active Aß immunotherapy designed to generate production of antibodies against a small Aß peptide fragment (Aß_1-6_) acting as a B-cell epitope avoiding an Aß-specific T-cell response	Safety, tolerability and immunogenicity of vaccine	During the core studies, AEs occurred in 35 patients (74.5%) treated with CAD106 and seven patients (63.6%) who received placebo. In the extension studies, the majority of patients reported at least one adverse event over the 66-week duration. Most AEs were mild to moderate in intensity. One patient died during the extension study.	Two patients discontinued during the extension studies due to serious adverse events.	Thirty CAD106-treated patients (63.8%) were serological responders.
Schneeberger 2015 [29]	A peptide-KLH conjugate vaccine where the peptide moiety mimics the N-terminal region of human Aß and designed to elicit Aß aggregate specific antibodies.	Safety, tolerability and immunogenicity of vaccine	A total of 210/233 patients in vaccine arm and 88/99 patients in control are had at least one adverse event. Out of them 14 patients in vaccine arm and 12 in control arm had serious AEs. Four deaths were reported during the trial, none considered related to treatment.	Three patients in vaccine arm and three in control arm discontinued the trial because of adverse events.	Vaccine induced IgG type antibodies to aggregated Aß, AD02 and KLH. However, a robust IgG antibody response was detected to KLH and AD02 and the antibody response to aggregated Aβ was low.
Ketter 2016 [30]	Active peptide vaccine against N-terminus 1-7 of Aß peptide	Safety, tolerability and immunogenicity of vaccine along with change in brain amyloid burden	Overall, ≥93% of patients in each treatment group experienced at least 1 treatment emergent adverse event, mostly mild to moderate in severity. Two deaths were reported in treatment group but were not considered to be related to study medication.	A total of 13 patients discontinued early from the trial, 11 from vaccine arm and two from control arm, due to treatment emergent adverse events.	More than 90% of the patients in the vaccine arm and none in the placebo arm were considered serological responders as per the predefined criteria.
Pasquier 2016 [31]	Active peptide vaccine against N-terminus 1-7 of Aß peptide	Safety, tolerability and immunogenicity of vaccine	Treatment emergent AEs due to any cause occurred in 92.6% of the patients. AEs in 3.3% of the patients were serious and treatment related. Two deaths, one in the vaccine arm and one in control arm, occurred during the study but none related to the study treatment.	Adverse events resulting in discontinuation or withdrawal from the study occurred in 7.4% of the participants.	All of the treatment groups achieved clear geometric anti- Aß IgG titer increases from baseline after 2 weeks compared to placebo where the response was not seen.
van Dyck 2016 [32]	Active peptide vaccine against N-terminus of 1-7 Aß peptide	Safety, tolerability and immunogenicity of vaccine along with change in brain amyloid burden	All but two of the participants developed treatment emergent AEs. A total of eight participants in vaccine arm and six in placebo arm developed serious treatment emergent AEs. AEs with fatal outcome were reported in none of the patients.	One patient withdrew from the trial because of adverse event from treatment, five due to loss of willingness to participate and one due to insufficient clinical response. 51/63 completed the study for 104 week, reason for discontinuation being non longer willing to participate in study.	Overall, 97.6% of the participants in the vaccine arm and none in the placebo arm met a predefined criteria for serologic response.
Hüll 2017 [33]	Active peptide vaccine against N-terminus 1-7 of Aß peptide	Safety, tolerability and immunogenicity of vaccine	Treatment emergent AEs were reported in 91.2% (145/159) and 88.7% (47/53) of subjects in EU/US and Japan studies respectively; AEs in 39.6% (EU/US) and 67.9% (Japan) of those subjects were assessed to be treatment-related. Only 3.1% (EU/US) and 11.3% (Japan) of subjects had serious treatment related AEs. Three deaths occurred in EU/US study and one in Japan study, none considered treatment related.	Adverse events leading to withdrawal from treatment or the study occurred in 8.8% of subjects in the EU/US studies and 15.1% in the Japan study	Higher anti-Aß IgG titers were observed in subjects who had received QS-21 + ACC-001 during the parent studies regardless of dose compared with those who had received ACC-001 alone or placebo during the parent studies.
Vandenberghe 2017 [34]	An active Aß immunotherapy designed to generate production of antibodies against a small Aß peptide fragment (Aß_1-6_) acting as a B-cell epitope avoiding an Aß-specific T-cell response	Safety, tolerability and immunogenicity of vaccine	The incidence of AEs was similar between vaccine and control arm (83% vs. 80%). Serious AEs were reported in 24.5% in vaccine arm and 6.7% in control arm. Three deaths in the vaccine arm reported, none related to the treatment.	A total of 10 patients in vaccine arm discontinued treatment due to adverse events or amyloid related imaging abnormalities.	CAD106 induced a strong Aß IgG response in 55.1% and 81.1% of the patients on 150 µg and 450 µg doses, respectively, versus none on placebo
Novak 2021 [35]	An active peptide vaccine against pathological tau protein	Safety, tolerability and immunogenicity of vaccine	AEs observed in 84.6% of AADvac1-treated individuals and 81.0% of placebo-treated individuals; serious AEs observed in 17.1% (vaccine arm) 24.1% (control arm) of patients. Two deaths reported on active treatment, none related to treatment.	A total of four patients in the vaccine arm and one in control arm discontinued the trial due to adverse events.	Serologic response rate as per the predefined criteria was 96.5% at the end of the initial six-dose regimen and 98.3% overall.

Overview of Ongoing Vaccination Trials

A systematic search on the website clinicaltrials.gov retrieved a single ongoing randomized, double blind, placebo controlled 24 months phase II study to investigate the safety, tolerability and immune response of repeated subcutaneous injections of ABvac40, an active vaccine against C-terminal end of Aß40 [36]. The trial is designed with an estimated enrollment of 120 patients, 55-80 years old with amnestic mild cognitive impairment or very mild AD. It was started in February 2018 and the estimated completion date was December 2022. Upon successful completion, the study aims to confirm the safety and tolerability data obtained from phase I clinical trial of ABvac40 by Lacosta et al. [24] Dosing schedule of ABvac40/placebo is six subcutaneous injections, the first five administered every four weeks and the sixth at week 42. Safety, tolerability and immune response, i.e., levels of anti-abeta40 antibodies in plasma are the primary outcome measures.

Discussion

While the early phase clinical trials for active immunotherapeutic approaches for AD have revealed promising results in terms of safety and immunogenicity, the therapeutic efficacy of the vaccines against AD is yet to be conclusively explored in adequately powered phase III studies. However, development of T-cell meningoencephalitis among 6% of patients who received AN-1792 in phase IIa clinical trial has led to the development of Aß targeting second generation vaccines designed to stimulate B-cell activation and antibody production, avoiding an Aß specific T-cell response [21,23,26,37]. In addition, even though the trial was interrupted with none of the participants receiving complete doses of the vaccine, long-term follow up of vaccinated patients, who were labelled as responders, maintained low but sustained, detectable antibody titers with significantly reduced functional decline [38]. These findings help emphasize the prospects of Aß immunotherapy as a potential means of reducing disease morbidity. Lastly, since the study population in all of the clinical trials consisted of elderly patients with AD, only a subset of reported adverse events were considered treatment related whereas majority of them fell into the spectrum of age-related comorbidities [20-35].

The immunogenicity of vaccines correlated with cumulative vaccine exposure with dramatic increase in antibody titers on subsequent immunization as compared to the titers after first injection of the vaccine [20-35]. Serological response rate of 100% was seen in four out of 16 included studies with study treatments UB-311 [23] and ACC-001 [27,31,33] whereas Gilman et al. [26] in the interrupted phase IIa trial for AN1792 reported a lowest serological response rate of 19.7%. Furthermore, studies also reported a sustained and higher antibody response with addition of adjuvants with study treatments like polysorbate-80 with AN1792 [20], QS-21 with ACC-001 [27,33] and CAD106 coupled with bacteriophage carrier protein [21].

The main limitation of this review is the characteristics of included studies. Assessments of safety and efficacy of the vaccines were made from phase I and phase II clinical trials with limited sample size and follow up duration. Since, none of the vaccines completed phase III trials, safety and efficacy assessments could not be confirmed with results of phase III trials with larger study population. With the inclusion of only interventional randomized or non-randomized clinical trials and exclusion of observational studies, comprehensive review of literature may have been impeded. Due to the differences in trial design along with formulation, dosage and administration of study treatments, comparative assessments of study outcomes could not be made. Furthermore, phase I clinical trials for three out of seven vaccines for AD (UB-311, AD02, ABvac40) were not followed up with subsequent phase II trials [23,24,29].

Despite the limitations of the study, this review serves to bridge the research gap by providing a systematic literature review and qualitative analysis of safety, tolerability and immunogenicity of early phase clinical trials, both ongoing and completed, for active immunotherapeutic efforts against AD. It is important to assert that continuous advancements in vaccine development with promising vaccine trials could not only help pioneer a safe and effective vaccine against AD, but it could also open doors to explore potential immunotherapeutic options for the wide spectrum of neurodegenerative disorders. However, attempts to develop vaccines against AD should always strive towards achieving highest therapeutic efficacy coupled with highest safety standards and cost-effectiveness. Large scale adequately powered clinical trials should, therefore, be conducted to detect rare adverse events associated with vaccination, explore changes in any potential disease biomarkers in vaccinated individuals along with the effect of host immune system competence to mount an immune response to vaccination. Furthermore, the role of anti-amyloid beta antibodies in non-AD tauopathies could also be explored. The authors are hopeful that clinical trials will pick up the pace in near future to further explore the role of vaccines against AD.

## Conclusions

This systematic review summarized the safety, tolerability, and immunogenicity of vaccines against AD where the majority of clinical trials reported promising results on all of the three outcome measures. This study also attempted to highlight the considerations which need to be heeded as one moves toward the development of active immunotherapeutic options for AD. Further high-quality phase III studies are needed to conclusively explore the clinical implications of active immunotherapies for AD patients.
